# Supplementary material for: SARS-CoV-2 Vaccine Effectiveness in Hospitalized Patients: A Multicenter Test-Negative Case–Control Study
Source: Vaccines (Basel). 2023 Nov 28;11(12):1779. doi: 10.3390/vaccines11121779 (PMC10747324; doi:10.3390/vaccines11121779)
Supplement: Supplementary file 1 [file vaccines-11-01779-s001.zip › vaccines-2697538-Supplementary Materials.pdf]

Table S1. Characteristics among controls, respiratory and non-respiratory.

	Respiratory Controls (324)	Non-Respiratory Controls (66)	*p*
Age (years)	52±17	50±19	0.43
Masculine gender (%)	58% (189)	42%(28)	0.01
BMI (kg/m^2)	27±7	27±5	0.95
At least one comorbidity (%)	74% (240)	62% (41)	0.27
Obesity	23% (75)	29% (19)	0.37
Diabetes	22% (72)	24% (16)	0.72
Hypertension	32% (103)	26%(17)	0.33
Cardiovascular disease	6% (20)	3% (2)	0.31
Current or previous tobacco smoking	48% (157)	38% (25)	0.11
Current tobacco smoking	9% (32)	6% (4)	0.61
Cigarettes/day	10±17	2±4	0.04
Biomass smoke exposure	31% (101)	42% (28)	0.08
At least one COVID-19 vaccine (%)	78% (254)	74% (49)	0.43
Full vaccination	63% (207)	59% (39)	0.46
Incomplete vaccination	14% (46)	13% (9)	0.90
1 dose	17% (54)	21% (14)	
2 doses	57% (186)	51% (34)	
3 doses	4% (14)	1% (1)	
Recent Travel	21% (69)	18% (12)	0.57
Self-reported adherence to protective measures	74% (239)	81% (54)	0.54
Personal risk	46% (149)	39% (26)	0.60
In Contact with SARS CoV-2 positive persons	12% (39)	7% (5)	0.29

Table S2. Different COVID-19 vaccines received by the participants.

	Cases with vaccination (191)	Controls with vaccination (301)
Pfizer BNT_{162b2} (Pfizer Inc./BioNTech)	23	65
AstraZeneca AZD1222 (AstraZeneca/Universidad de Oxford)	67	122
Sputnik V Gam-COVID-Vac (Instituto Gamaleya)	26	58
CanSino Ad5-nCoV (CanSino Biologics, Inc.)	16	12
SinoVac CoronaVac (SinoVac Research and Development Co.)	45	27
Moderna mRNA-1273 ModernaTX, Inc.	1	2
Janssen Ad26.COV2-S (Janssen-Cilag)	1	1
Does not know	12	14
